# BARRIERS AND FACILITATORS TO TECHNOLOGY USE AMONG RURAL CAREGIVERS OF PEOPLE LIVING WITH DEMENTIA

**DOI:** 10.1093/geroni/igae098.2520

**Published:** 2024-12-31

**Authors:** Sarah Boucher, Anna Jolliff, Matthew Zuraw, Christian Elliot, Nicole Werner

**Affiliations:** Indiana University Bloomington, Bloomington, Indiana, United States; Indiana University Bloomington, Bloomington, Indiana, United States; CareVirtue, San Diego, California, United States; CareVIrtue, San Diego, California, United States; Indiana University, Bloomington, Bloomington, Indiana, United States

## Abstract

Caregivers of people living with Alzheimer’s disease or related dementias (ADRD) who live in rural areas experience more difficulty in accessing resources to support caregiving. Technology may be a promising tool to connect rural caregivers to support. However, little is known about rural ADRD caregivers’ technology use. The objective of this study was to assess the barriers and facilitators to technology use among ADRD caregivers living in rural communities. In this qualitative study, rural caregivers of people living with ADRD were recruited through community partners to complete virtual semi-structured interviews. Interview questions focused on formal and informal supports used by caregivers and the role of technology in accessing those supports. Two coders completed an inductive, iterative thematic analysis. Caregivers (N=19) were 73.7% female and had a mean age of 66.4(SD=10.1). Within the sample, 57.9% of caregivers were caring for a partner, while 31.6% cared for a parent or parent-in-law. Analyses resulted in fourteen codes, which were combined into three themes. Barriers and facilitators to technology use included 1) systemic influences, such as access to technology and support with using it; 2) alignment between technologies available and caregivers’ informational needs; and 3) a preference for technological versus in-person support, including features of technology that influence this preference. The results of this study demonstrate systemic barriers that must be addressed when introducing technology-based interventions to rural caregivers. Further, results point to enhancements that can be made to technology-based interventions to improve their usefulness to rural caregivers.

